# Factors associated with overreporting based on community verification results in a performance-based financing program in Zimbabwe

**DOI:** 10.1186/s12913-025-12599-8

**Published:** 2025-05-28

**Authors:** Trina Gorman, Pia Arce, Gabrielle O’Malley, Taurai Kambeu, Brian Maponga, Jabulani Mavudze, Sinokuthemba Xaba, Getrude Ncube, Bernardo Hernandez

**Affiliations:** 1 Department of Global Health, University of Washington, Seattle, WA USA; 2 Gorman Consulting, Edmonds, WA USA; 3 School of Public Health, National Institute of Public Health of Mexico, Cuernavaca, Mexico; 4 Department of Health Metrics Sciences, University of Washington, Seattle, WA USA; 5 Population Services International, Harare, Zimbabwe; 6 The Ministry of Health and Child Care, Harare, Zimbabwe

**Keywords:** Community verification, Performance-based financing, Risk-based sampling, Overreporting, Patient verification

## Abstract

**Background:**

While most Performance Based Financing (PBF) programs perform community verifications to confirm patients received reported services, many focus analysis and payment calculations on facility record verification due to their lower cost. Risk-based sampling can reduce the cost of community verifications by targeting areas with the highest risk of overreporting but there is little research on the factors associated with risk to guide sampling decisions.

**Objective:**

This study explores facility-level and district-level factors associated with overreporting within a PBF setting.

**Methods:**

Using community verification data from a Voluntary Medical Male Circumcision (VMMC) program in Zimbabwe, we estimated two binary outcomes with generalized mixed effects models. Our primary outcome is a measure of overreporting, defined as when interviewed patients did not plausibly confirm receipt of the VMMC. Additionally, we assessed factors associated with patients who were selected but ultimately not interviewed. We employed inverse probability of treatment weighting to address non-response and bootstrapping-based multiple imputation to address missingness.

**Results:**

We found that patients in the target age range, which were compensated at a higher price point, were less likely to be interviewed and over two times more likely to be classified as overreported compared to patients outside this age range (OR: 2.92, 95% CI: 2.38–3.59). Patients from outside the fixed health facility were more likely to be interviewed and less likely to be classified as overreported. In-person interviews as opposed to phone interviews appeared to be a worthwhile investment (OR: 1.61, 95% CI: 1.20–2.16).

**Conclusion:**

We identified various factors that were associated with unsubstantiated VMMCs to inform risk-based sampling; however, our findings also suggest potential data fabrication. Programs should consider employing similar methods to reduce costs and increase the use of community verification data.

**Supplementary Information:**

The online version contains supplementary material available at 10.1186/s12913-025-12599-8.

## Introduction

Performance- based financing (PBF) has generated substantial interest among governments and funders in the last 15 years [1]. The theory that underpins PBF is that aligning provider payment with provided services increases health care worker productivity and service quality. Independent verification is an established cornerstone of PBF programs to ensure that reported outcomes, corresponding payments, and estimated public health benefits are accurate.

Typically, programs store aggregated reported outcome totals in a health management information system (HMIS), totaling the number of services reflected in records (often paper-based) at health facilities. To verify the reported totals, PBF programs typically include reviews of facility records (also called quantity verification) and interviews with patients (called community verification, counter verification, or client tracing) [2]. Ultimately, these activities aim to identify and reduce overreporting, which can be either intentional (e.g. workers fabricating data to increase their pay) or unintentional (e.g. bookkeeping errors due to insufficient time or training). Because overreporting can be caused by issues in the aggregation process or by inaccuracies about the service itself, it’s important for verifications to be explicit about the source data used to compare the reported totals against. For quantity verifications, overreporting is estimated *based on facility records* (e.g., when the number of services in the HMIS exceed the number substantiated in records). For community verifications, overreporting is estimated *based on patient responses* (e.g., when a patient’s report about a service does not match the record). Measuring both types of overreporting is important to ensure stakeholders can trust the outcomes; the former helps improve recordkeeping and the latter ensures records represent real delivered services (e.g. the validity of records)—a critical component of PBF given the same incentives that are intended to promote productivity can also entice intentional overreporting.

Even though PBF programs acknowledge the importance of confirming services were received and invest significant resources in community verification [1–4], the literature suggests that results from these activities are commonly not analyzed program-wide or used to adjust payment. Among eight case studies that included community verifications [5–12], five explicitly mention the lack of data use of the community verification results [5, 9–12]. Instead, quantity verification data are often relied on because community verification is more costly and time-intensive per service verified [5]. Costs may be reduced by targeting areas empirically most prone to overreporting, also called risk-based sampling [5, 13–16]. To implement risk-based sampling, program planners need to know which services to oversample but there are no known studies that assess factors associated with overreporting based on community verification results (or other ways to measure the validity of records). The only other known study on the topic used facility records as the source data [17].

This study contributes to closing this gap by assessing facility-level and patient-level factors associated with overreporting, where overreporting means the patients was unable to plausibly confirm receipt of the service, using data from a PBF program designed to scale VMMC in Zimbabwe. Findings from these analyses can help implementers better target community verifications, and ultimately make them more sustainable.

## Methods

### Study setting and PBF program

The incidence of HIV/AIDs in Zimbabwe in 2009 was 669 new cases per 100,000 people – an estimate that has dramatically improved to 214 new infections per 100,000 people per year in 2019 [18]. Part of this progress was due to the VMMC program, which the Ministry of Health and Child Care (MoHCC) launched in 2009. In 2015, the Bill & Melinda Gates Foundation began supporting Population Services International (PSI) to help scale the program using PBF. This study focuses on VMMCs reported between May 2016 and April 2019 as part of a grant. VMMCs were performed at VMMC locations, which were either fixed health facilities or locations staff traveled to on outreach. Mobilizers, categorized as gold or silver based on performance, sensitized communities and enroll interested patients across their assigned district. PSI and VMMC staff were paid on a per-unit basis for each VMMC performed. Patients between 15–29 years old were in the target age range, so PSI as an organization and mobilizers were renumerated at a higher price point for this group. The VMMC outcomes were aggregated into the HMIS each month, which is the data source used for verification as outlined below.

### Verification design

**Fig. 1:**
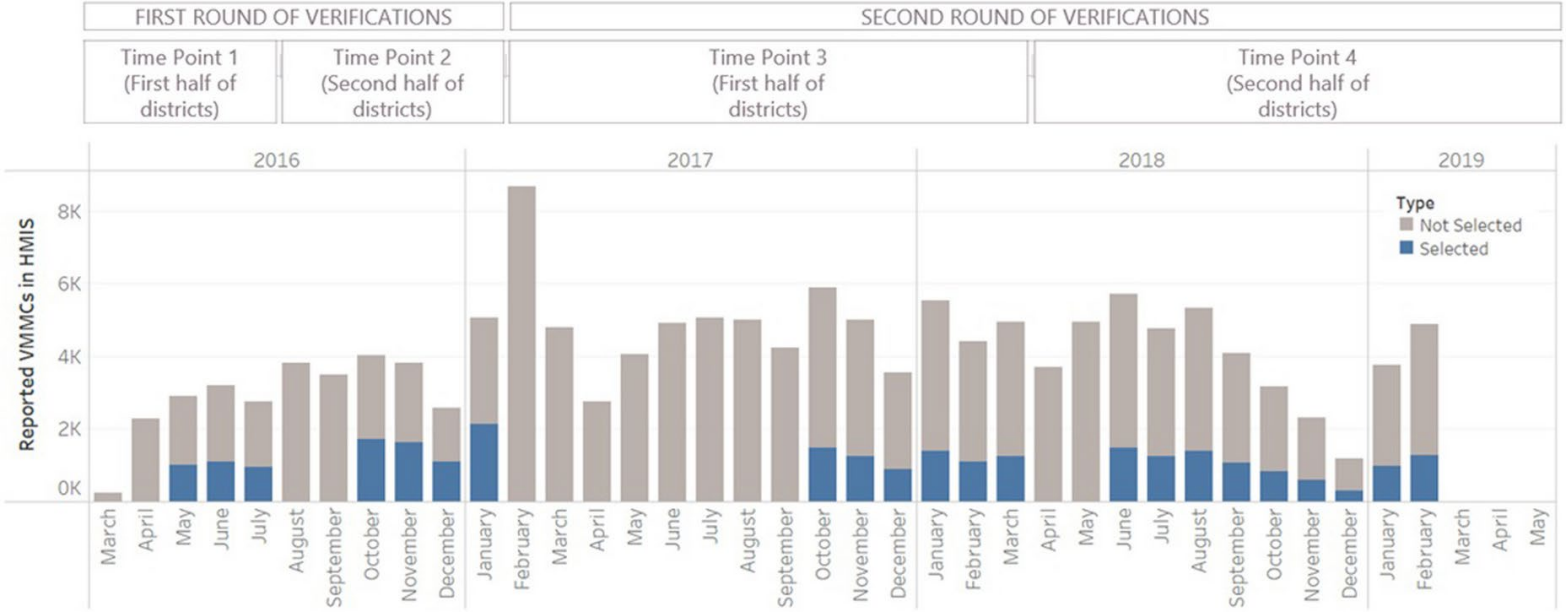
Sample of facility records included in this analysis, from which patients were randomly drawn

Verification teams performed quantity verifications and community verifications at four time points, with two rounds of verification for each district in the program (Fig. 1). For the quantity verification, the alignment between the number of sufficiently complete facility records at VMMC locations and the number of reported VMMCs in the HMIS was assessed. For community verification, patients were interviewed to assess the alignment between patients’ reports of receiving the service and sufficiently complete facility records from the quantity verification. Patients selected for community verification were interviewed on the phone, if available, or traced to their home. To confirm receipt of the VMMC, patient responses (or in the case of minor’s, their parent’s responses) had to plausibly match four fields on the facility record: patients were asked if they were circumcised, and if they were, they were asked the general time period, district, and VMMC method (surgery of prepex device). This helped ensure that the patient’s VMMC uniquely matched the selected record. Patient responses were compared after the interviews with data extracted from the facility record. If one or more of these four requirements did not align, the VMMC outcome was considered overreported.

PSI selected a purposeful sample of records and from the sample of records, they selected patients. The funder required that among reported VMMCs, 25% of records needed to be reviewed and 2.5% of patients needed to be interviewed. To select the sample records, the team divided the districts in half so that all districts were visited once per verification round; VMMC locations in the first half could be selected for the first and third verifications and VMMC locations in the second half could be selected in the second and fourth verifications. To select the portion of months to verify, PSI selected between 3 and 9 months of VMMCs prior to each time point in order to be on track to reach the 25% requirement. Within these months, VMMC locations that reported above 50 VMMCs and were from the relevant districts were selected for quantity verification. To select the sample of patients for community verification, 10% of sufficiently complete facility records were randomly selected.

### Dataset for this study

**Fig. 2:**
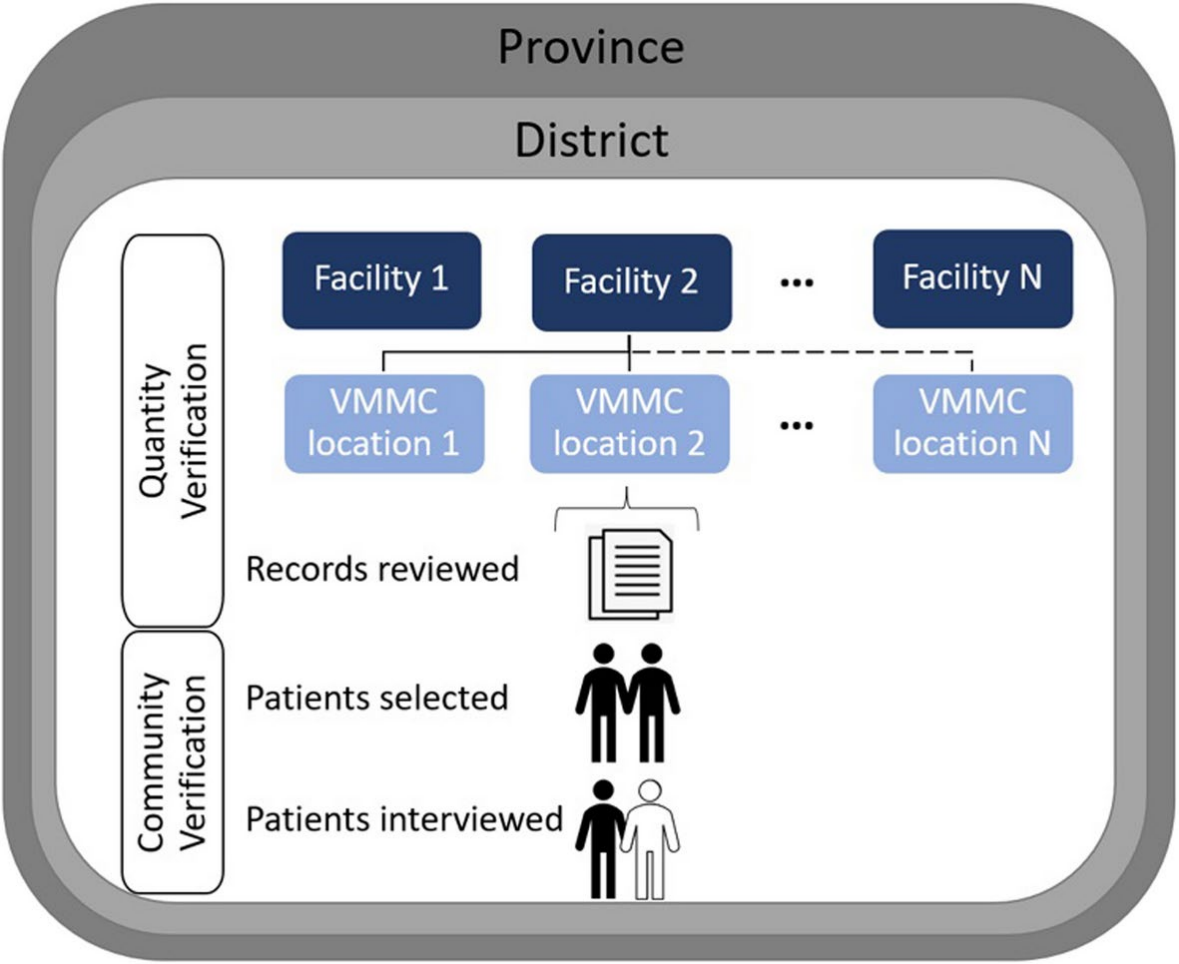
Data structure

This study conducted secondary analysis using data from the program activities described above as well as data for the explanatory variables provided separately by the MoHCC. PSI provided a dataset at the patient-level; patients were nested within the VMMC locations where the procedures took place, the fixed health facility (whose staff performed the procedures), districts and provinces (Fig. 2). For each patient, there were three sources of data. First, data that the verification team had extracted from facility records including the requirements to match with patient responses. Second, data from tracking sheets documenting whether the patient was found for the interview and if not, the reason. Third, the results from the community verification survey for the subset that were found and interviewed. For the explanatory variables, a list was created of the variables of interest based on existing literature, after which PSI and the MoHCC collected the variables that were available from district-level VMMC officials (Table 1). Patient-level characteristics were also included as explanatory variables. Our hypothesis was that a later verification round would be negatively associated with overreporting and that distance would be negatively associated with being interviewed, and positively associated with overreporting.

**Table 1:** Summary of explanatory variables

Level	Variables
District	Largest number of gold mobilizers that were active at one time during the grant period
	Largest number of silver mobilizers that were active at one time during the grant period
	Largest number of VMMC vehicles operating at one time during the grant period
	Whether it was the first or second round of verifications for the district
Health Facility	Number of VMMCs reported in the HMIS for the health facility at all VMMC locations throughout the grant period
	Largest number of trained VMMC staff (nurses & doctors) that were employed at one time during grant period
VMMC Location	Number of supervisory visits provided by the MOHCC
	Size of eligible population in catchment area
	Distance in kilometers from the VMMC location to the health facility based on GPS coordinates
	Type of facility where the VMMC took place (e.g., primary, secondary, tertiary, central care)
	Type of base funding where the VMMC took place (government, local council, mission, private)
Patient	Age of patient from the facility record
	VMMC method (prepex or surgical) from the facility record
	Whether the patient was interviewed on the phone or at home

Some VMMC locations in the program data were not selected for two verifications. To eliminate bias caused by the influence of the location being verified a second time, the dataset was restricted to only include the first verification from each location, though the dataset contains some repeated measures at the health facility level. Figure 1 summarizes the purposeful sample of facility records, from which patients were randomly sampled for this analysis.

### Analysis

The purpose of this secondary data analysis was to examine the factors associated with overreporting, where overreporting means a service in a complete facility record was not plausibly received by the patient. Two binary outcomes were assessed using two models. The first outcome indicated when patients were not interviewed (yes for not interviewed; no for interviewed) among patients selected for community verification (Model 1). The second outcome indicated when patients did not meet the funders four requirements to confirm receipt (yes for did not meet requirements; no for did meet) among patients that were interviewed (Model 2). For both outcomes, a generalized linear mixed effects model was used, including a random intercept for the health facility and the district to account for the nested structure of the data. Both models used the binomial family and logit link. The models were also stratified to assess whether findings differed between high/low performing facilities or between facilities with large/small catchment populations. For the former, facilities were classified as high performing if they reported more than the median number of VMMCs over the course of the grant period, and low otherwise. Facilities were similarly classified as having large catchment populations if they had more than the median population across sampled facilities, and low otherwise.

To account for patients that were selected but not interviewed (Model 1), inverse probability of treatment weighting (IPTW) was employed, using the explanatory variables that appeared to differentiate the interviewed from the not interviewed based on a t-test. Among the variables in Table 1, those that were not used included the number of VMMC staff and the number of supervisory visits. To reduce the variance of the effect estimate, the weights were stabilized and then trimmed at 1st and 99th percentiles. To address missingness in three variables (catchment population, number of supervisory visits, VMMC method) bootstrapping-based expectation–maximization multiple imputation was used. Five datasets were imputed separately for location level variables and patient level, each including all relevant independent variables as predictors. After combining the location-level and patient-level datasets, regressions were run separately on each of the imputed datasets and then the coefficients and standard error estimates were pooled using Rubin’s rules [19]. To test the sensitivity of the findings to missing data, all models were also estimated using only observations that had no missing data (referred to as complete case analysis). More details about the weighting and imputation can be found in Appendix 1 and Appendix 2.

All analyses were performed in STATA version 17.0 (StataCorp. 2023. Stata Statistical Software: Release 17. College Station, TX: StataCorp LLC.) or R version 2022.12.0 (R Foundation for Statistical Computing, Vienna, Austria). Multiple imputation was performed using the Amelia II package version 1.8.1 [20].

## Results

Two thousand five hundred sixty-five records were selected for community verification and included in the sample. Among them, 45% (1153/2565) of patients were not interviewed. Among interviewed patients, 19% (263/1412) were classified as overreporting. The exclusion of individuals from repeated verification rounds is the reason why this sample is smaller than in our previous analysis (Gorman T, Hernandez B, Garnett G, Erhard L, Kambeu T, Maponga B, et al: A results to action framework for community verification: a case study from performance based financing program in Zimbabwe, unpublished).

### Characteristics of patients selected for community verification

Table 2 describes the sample of patients. Among all selected patients, the majority were circumcised surgically (89%), and in the target age range 15–29 years old (58%); 62% were selected as part of the second round of verifications. Patients were from health facilities that on average reported 5658 VMMCs and had 5 clinical staff. 52% of patients were circumcised outside of the fixed health facility but within 50 km. The average catchment population of the VMMC location was 12,425. Comparing selected patients with those that were interviewed, the largest differences included that interviewed patients were from facilities with lower reported VMMCs (5,187), smaller catchment populations (11,080), and had a greater proportion of patients below 15 years old (47%). Appendix 3 describes the sample of patients that were included in the complete case analysis.

**Table 2:** Description of patients

	Patients selected for community verification					Patients interviewed for community verification				
	Mean or Proportion	SD	Min	Max	N	Mean or Proportion	SD	Min	Max	N
Number of reported VMMCs (Log)	5658.2	3650.9	22	14,829	2565	5187.2	3278.5	22	14,829	1412
Number of staff	5.436	2.088	2	10	2565	5.475	2.044	2	10	1412
Number of gold mobilizers	4.239	0.991	1	6	2565	4.277	0.967	1	6	1412
Number of silver mobilizers	4.05	4.154	0	15	2565	3.772	3.882	0	15	1412
Number of vehicles	1.795	0.727	1	3	2565	1.697	0.738	1	3	1412
Population of catchment	12,425.2	20,568.1	590	156,842	2523	11,080.3	21,206.7	590	156,842	1389
Location distance from facility^*^										
[0]	0.412	0.492	0	1	2565	0.395	0.489	0	1	1412
[> 0 to 50]	0.526	0.499	0	1	2565	0.539	0.499	0	1	1412
[> 50 to 150]	0.062	0.24	0	1	2565	0.066	0.248	0	1	1412
Number of supervisory visits	2.532	2.048	0	10	2268	2.524	2.135	0	10	1350
Type of VMMC location^*^										
District	0.194	0.396	0	1	2565	0.233	0.423	0	1	1412
Mission	0.142	0.349	0	1	2565	0.161	0.367	0	1	1412
Rural clinic	0.562	0.496	0	1	2565	0.541	0.498	0	1	1412
Rural hospital	0.102	0.303	0	1	2565	0.065	0.247	0	1	1412
Type of base funding^*^										
Council	0.313	0.464	0	1	2565	0.288	0.453	0	1	1412
Government	0.465	0.499	0	1	2565	0.482	0.5	0	1	1412
Mission	0.207	0.405	0	1	2565	0.21	0.407	0	1	1412
Private	0.014	0.118	0	1	2565	0.021	0.144	0	1	1412
Verification round for district^*^										
First	0.388	0.487	0	1	2565	0.409	0.492	0	1	1412
Second	0.612	0.487	0	1	2565	0.591	0.492	0	1	1412
Age of Patient^*^										
Less than 15	0.374	0.484	0	1	2565	0.47	0.499	0	1	1412
15-29 years	0.583	0.493	0	1	2565	0.489	0.5	0	1	1412
30 or more	0.043	0.202	0	1	2565	0.041	0.199	0	1	1412
Method^*^										
Prepex	0.111	0.314	0	1	2512	0.13	0.337	0	1	1396
Surgical	0.889	0.314	0	1	2512	0.87	0.337	0	1	1396
Interview Location^*^										
On phone						0.461	0.499	0	1	1412
In person						0.539	0.499	0	1	1412

^*^Every category is shown as a dummy where the variable equals 1 and 0 otherwise

### Factors associated with a patient that was not interviewed (Model 1)

Table 3 presents the findings for factors associated with when a patient was not interviewed after controlling for the other facility and district level variables in the table. Relative to VMMCs performed at a fixed health facility, patients whose procedures took place any distance away were more likely to be interviewed; considering the stratified analysis, this appears to have been driven by facilities with large catchment populations (Appendix 4). Older patients and patients who had a surgical procedure (OR: 2.21, 95% CI: 1.57–3.12) were both more likely to not be interviewed. In particular, patients that were in the target age range of 15–29 years had the highest association, resulting in nearly three times the likelihood of not being interviewed (OR:2.92, 95% CI: 2.38–3.59); this group was positively associated with overreporting in each of the stratified models, suggesting the size of the catchment or number of reported did not influence this outcome. The complete case analysis additionally showed that locations with larger catchment populations were negatively associated with not being interviewed (OR:0.81, 95% CI: 0.66–0.99) (i.e. are more likely to be interviewed in a large catchment), and patients from the second round of verifications were positively associated (OR:1.88, 95% CI: 1.27–2.80).

**Table 3:** Logit regression results for not being interviewed (Model 1)

	Results with Imputed Values (*N* = 2,565)					Complete Case Analysis (*N* = 2,214)				
	Adj. Odds ratio*	Std. err	t	[95% conf. interval]		Adj. Odds ratio*	Std. err	t	[95% conf. interval]	
Number of reported VMMCs (Log)	1.38	0.23	1.89	0.99	1.92	1.34	0.24	1.62	0.94	1.90
Number of staff	1.13	0.09	1.55	0.97	1.33	1.10	0.07	1.37	0.96	1.25
Number of gold mobilizers	0.83	0.15	−1.00	0.58	1.19	0.88	0.12	−0.99	0.68	1.14
Number of silver mobilizers	1.00	0.05	−0.04	0.90	1.10	1.05	0.04	1.37	0.98	1.13
Number of vehicles	1.56	0.49	1.42	0.84	2.88	1.31	0.31	1.16	0.83	2.08
Population of catchment (Log)	0.98	0.09	−0.26	0.82	1.16	**0.81**	**0.08**	**−2.07**	**0.66**	**0.99**
Location distance (km)										
[0]	(base)	(base)	(base)	(base)	(base)	(base)	(base)	(base)	(base)	(base)
[> 0 to 50]	**0.62**	**0.15**	**-2.03**	**0.39**	**0.98**	0.83	0.22	−0.70	0.50	1.39
[> 50 to 140]	**0.35**	**0.11**	**-3.39**	**0.19**	**0.64**	**0.48**	**0.16**	**-2.18**	**0.25**	**0.93**
Number of supervisory visits	0.90	0.05	-1.76	0.80	1.02	0.99	0.05	−0.18	0.90	1.09
Type of facility										
District	(base)	(base)	(base)	(base)	(base)	(base)	(base)	(base)	(base)	(base)
Mission	0.78	0.25	-0.76	0.42	1.46	0.90	0.29	−0.31	0.48	1.71
Rural clinic	1.05	0.29	0.20	0.62	1.80	0.68	0.19	-1.41	0.40	1.16
Rural hospital	1.45	0.42	1.29	0.82	2.56	0.71	0.23	-1.05	0.37	1.35
Type of base funding										
Council	(base)	(base)	(base)	(base)	(base)	(base)	(base)	(base)	(base)	(base)
Government	1.10	0.16	0.63	0.82	1.46	1.31	0.20	1.73	0.96	1.77
Mission	1.09	0.28	0.34	0.66	1.81	0.95	0.25	-0.19	0.57	1.60
Private	0.46	0.25	-1.44	0.16	1.33	0.70	0.37	-0.67	0.25	1.97
Verification round										
First	(base)	(base)	(base)	(base)	(base)	(base)	(base)	(base)	(base)	(base)
Second	1.14	0.20	0.74	0.81	1.61	**1.88**	**0.38**	**3.13**	**1.27**	**2.80**
Age of Patient										
Less than 15	(base)	(base)	(base)	(base)	(base)	(base)	(base)	(base)	(base)	(base)
15-29 years	**2.92**	**0.31**	**10.25**	**2.38**	**3.59**	**2.99**	**0.33**	**9.87**	**2.41**	**3.72**
30 or more	**2.09**	**0.51**	**3.04**	**1.30**	**3.37**	**2.31**	**0.65**	**2.97**	**1.33**	**4.01**
Method										
Prepex	(base)	(base)	(base)	(base)	(base)	(base)	(base)	(base)	(base)	(base)
Surgical	**2.21**	**0.39**	**4.56**	**1.57**	**3.12**	**2.29**	**0.44**	**4.32**	**1.57**	**3.34**

All odds ratios are adjusted by all the variables in the table

### Factors associated with overreporting (Model 2)

Table 4 presents findings for factors associated with overreporting after including the same variables as in Model 1. Relative to VMMCs performed at a fixed health facility, procedures that took place away were less likely to be classified as overreporting as were procedures that took place as part of the second round of verifications (OR: 0.39, 95% CI: 0.25–0.61). Overreporting was more likely for fixed health facilities that reported greater numbers of VMMCs (OR: 2.00, 95% CI: 1.16–3.44) and VMMC locations that were rural hospitals (OR: 3.28, 95% CI: 1.28–8.40). In terms of patient level characteristics, overreporting was more likely among patients in the target age range (OR: 2.21, 95% CI: 1.49–3.26) and when the patient was interviewed in person (OR: 1.61, 95% CI: 1.20–2.16). Considering the stratified analyses, these results appeared to be largely driven by facilities that reported more VMMCs given each significant association held when the regression was run among patients from these facilities (Appendix 5). The stratified analyses also showed that patients from the target age range were not associated with overreporting among facilities with small catchment areas, but the positive association held in facilities with large populations as well as facilities with both small/large reported VMMCs. And finally, the complete case analysis additionally showed that the number of gold mobilizers and catchment population were negatively associated with overreporting and surgical procedures were positively associated; location distance and rural hospitals were also not significant in the model with only complete data.

**Table 4:** Logit regression results for overreporting defined by when a patient did not plausibly confirm receipt (Model 2)

	Results with Imputed Values (*N* = 1,412)						Complete Case Analysis (*N* = 1,334)					
	Adj. Odds ratio*	Std. err	t	P >|t|	[95% conf. interval]		Adj. Odds ratio*	Std. err	t	P >|t|	[95% conf. interval]	
Number of reported VMMCs (Log)	**2.00**	**0.55**	**2.51**	**0.012**	**1.16**	**3.44**	1.59	0.44	1.68	0.094	0.92	2.72
Number of staff	1.02	0.07	0.30	0.761	0.90	1.16	1.04	0.07	0.58	0.560	0.91	1.19
Number of gold mobilizers	0.83	0.08	−1.88	0.061	0.68	1.01	**0.84**	**0.07**	**−2.14**	**0.032**	**0.72**	**0.99**
Number of silver mobilizers	0.99	0.04	−0.26	0.791	0.91	1.07	1.02	0.03	0.53	0.595	0.96	1.08
Number of vehicles	0.81	0.22	−0.77	0.440	0.47	1.39	0.81	0.14	−1.19	0.235	0.57	1.15
Population of catchment (Log)	0.91	0.07	−1.22	0.221	0.77	1.06	**0.87**	**0.06**	**−2.10**	**0.036**	**0.77**	**0.99**
Location distance (km)												
[0]	(base)	(base)	(base)	(base)	(base)	(base)	(base)	(base)	(base)	(base)	(base)	(base)
[> 0 to 50]	**0.41**	**0.11**	**-3.28**	**0.001**	**0.24**	**0.70**	0.80	0.28	-0.64	0.525	0.40	1.59
[> 50 to 140]	**0.36**	**0.15**	**-2.42**	**0.016**	**0.16**	**0.82**	0.74	0.23	-0.95	0.340	0.40	1.38
Number of supervisory visits	0.91	0.06	-1.47	0.141	0.79	1.03	0.96	0.05	-0.81	0.416	0.87	1.06
Type of VMMC location												
District	(base)	(base)	(base)	(base)	(base)	(base)	(base)	(base)	(base)	(base)	(base)	(base)
Mission	1.28	0.62	0.51	0.612	0.49	3.32	0.84	0.46	-0.33	0.742	0.29	2.44
Rural clinic	1.63	0.57	1.40	0.162	0.82	3.24	0.93	0.38	−0.17	0.869	0.42	2.08
Rural hospital	**3.28**	**1.57**	**2.48**	**0.013**	**1.28**	**8.40**	1.83	0.85	1.30	0.195	0.73	4.54
Type of base funding												
Council	(base)	(base)	(base)	(base)	(base)	(base)	(base)	(base)	(base)	(base)	(base)	(base)
Government	0.91	0.21	−0.43	0.667	0.58	1.42	1.11	0.27	0.41	0.680	0.69	1.78
Mission	0.62	0.16	−1.85	0.064	0.38	1.03	0.78	0.17	-1.13	0.257	0.51	1.20
Private	0.27	0.21	-1.66	0.097	0.06	1.27	0.37	0.26	-1.39	0.163	0.09	1.50
Verification round												
First	(base)	(base)	(base)	(base)	(base)	(base)	(base)	(base)	(base)	(base)	(base)	(base)
Second	**0.39**	**0.09**	**−4.17**	**0.000**	**0.25**	**0.61**	**0.34**	**0.11**	**−3.25**	**0.001**	**0.18**	**0.65**
Age of Patient												
Less than 15	(base)	(base)	(base)	(base)	(base)	(base)	(base)	(base)	(base)	(base)	(base)	(base)
15-29 years	**2.21**	**0.44**	**3.96**	**0.000**	**1.49**	**3.26**	**2.21**	**0.47**	**3.74**	**0.000**	**1.46**	**3.34**
30 or more	1.15	0.51	0.31	0.755	0.48	2.74	1.34	0.65	0.61	0.541	0.52	3.45
Method												
Prepex	(base)	(base)	(base)	(base)	(base)	(base)	(base)	(base)	(base)	(base)	(base)	(base)
Surgical	1.81	0.58	1.84	0.066	0.96	3.39	**2.21**	**0.66**	**2.67**	**0.008**	**1.23**	**3.96**
Interview Location												
On phone	(base)	(base)	(base)	(base)	(base)	(base)	(base)	(base)	(base)	(base)	(base)	(base)
In person	**1.61**	**0.24**	**3.17**	**0.002**	**1.20**	**2.16**	**1.62**	**0.29**	**2.66**	**0.008**	**1.13**	**2.30**

All odds ratio are adjusted by all the variables in the table

## Discussion

To our knowledge, this is the first study to quantitatively estimate the factors associated with not being interviewed and overreporting based on community verification results in a PBF setting. While both outcomes indicate that a facility record was not confirmed, the latter is of most interest to PBF funders since the errors leading to discrepant interviews are more often in the facility’s control.

We found that patients in the target age range, which were renumerated at a higher price point, were more likely to both not be interviewed and be classified as overreported. While this finding could be used to inform risk-based sampling, which was the primary purpose of our analysis, it importantly also suggests potential data fabrication—particularly since it aligns with suspicions from the verifiers who performed the fieldwork as presented in a previous study for this same project (Gorman T, Hernandez B, Garnett G, Erhard L, Kambeu T, Maponga B, et al: A results to action framework for community verification: a case study from performance based financing program in Zimbabwe, unpublished). Some verifiers suspected that staff fabricated information on the facility records to increase their pay. Verifiers explained that a common cause of an unconfirmed VMMC by a patient was when a patient had signed up with a mobilizer but did not go through with the procedure; since mobilizers were paid more for this age group, there could have been opportunistic completion of records following the initial signup. This finding also aligns with evidence from a series of in-depth case studies on the unintended consequences of PBF for a Burkina Faso PBF program. Turcotte-Tremblay et. al. found that in two [11, 21] out of their three cases studies [11, 21, 22], there was widespread data fabrication by staff whose payment was conditioned on results in order to increase their pay. Program planners who are in charge of the design of PBF programs should consider increasing the resources invested in community verifications—the only commonly used verification method that can detect fraud. In particular, it is concerning that community verification results are often not analyzed [9, 12] or used to inform payment [5, 10, 11]; if fabrication is indeed happening in these programs, there would be no way to detect it and large sums of funding would be paid out in error.

It was surprising that patients who were circumcised away from the fixed health facility were more likely to be interviewed. We expected the opposite, given that finding such patients requires more resources [5], and some verifiers reported that they had insufficient time and resources to exhaustively look for all patients (Gorman T, Hernandez B, Garnett G, Erhard L, Kambeu T, Maponga B, et al: A results to action framework for community verification: a case study from performance based financing program in Zimbabwe, unpublished). The fixed health facilities were often in district centers, so one possible explanation is that outside villages were smaller, making it easier for villagers to assist with locating patients.

The second round of verifications at the district level was associated with a decreased likelihood of overreporting, which is in line with various other studies that have shown that audits improve data quality [23, 24]. While the VMMC locations and patients had no repeated measures in our dataset, the VMMC staff from health facilities could have either been involved in an earlier verification and/or knew the verifications had occurred and as a result, improved record keeping and/or reduced any data fabrication. Finally, interviews performed in person were more likely to identify overreporting, suggesting tracing patients to their homes when they were not reachable by phone was worth the additional resources. This could be due to the verifier’s improved ability to gain rapport and probe in person [11].

This study is part of an effort to better target and reduce the cost of PBF verifications. Reducing the cost of community verifications is of particular importance since an eyewitness account from a patient is a more valid data source than documentation created by staff who benefit when more services are reported. Without cost-effective solutions, community verifications could be deprioritized, as shown by the suspension of these efforts in Benin [5]. There is also emerging evidence that quantity verifications can portray a misleading and overly positive assessment of the data quality of reported VMMCs [11], (Gorman T, Hernandez B, Garnett G, Erhard L, Kambeu T, Maponga B, et al: A results to action framework for community verification: a case study from performance based financing program in Zimbabwe, unpublished), [21]. Community verifications shed light on the extent to which reported VMMCs and the resulting health impact were actually realized. To reduce their cost, we identified factors associated with unconfirmed services, which programs could consider during risk-based sampling decisions.

This study has several limitations. Patients were a random sample from a purposeful sample of VMMC locations and months, so may not be representative of all reported VMMCs in the grant period. In particular, facilities that reported fewer VMMCs were not included in the sample, though we were still able to detect an association between the number of reported VMMCs and overreporting. Although our analysis was able to control for various facility and geographical characteristics, other unmeasured variables may confound the relationship between these factors and the two outcomes. In particular, we were unable to look at whether facilities with higher workloads (e.g., size of catchment population per staff) tended to have more unsubstantiated VMMCs although catchment size and number of staff were included separately in each model. In addition, regarding the association identified between patients in the target age range and both outcomes; while one explanation is data fabrication as suspected by the verifiers, other confounder factors could be correlated with the target age range such as stigma (e.g., if older patients were more likely to report false information to avoid follow-up). In terms of generalizing to other contexts, while these findings are likely to be unique to this context, other programs could consider similar analyses to guide sampling and programmatic decisions. In particular, including factors that are tied to payment in analyses could help detect potential data fabrication.

## Conclusion

Analyzing community verification data can inform future risk-based sampling as a step toward reducing the cost and increasing the sustainability of these efforts. Importantly however, community verification data are also essential to detect data fabrication, which we unexpectedly identified in these analyses. More in-depth community verifications and analyses of the resulting data are needed within PBF programs to ensure payment is tied to accurate outcomes.

## Supplementary Information

Supplementary Material 1.

## Figures and Tables

**Fig. 3:**
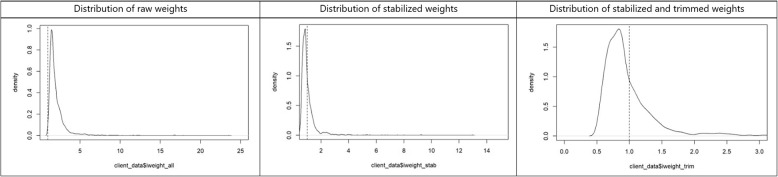
Distribution of weights

**Fig. 4:**
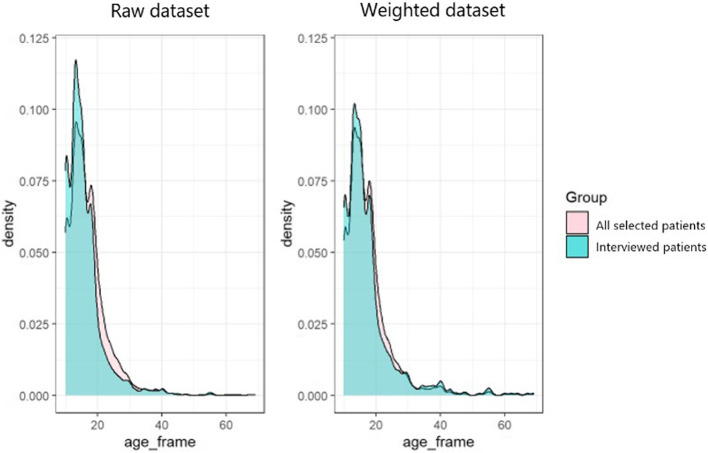
Distribution of the age of patients, comparing the raw data and the weighted data

**Fig. 5:**
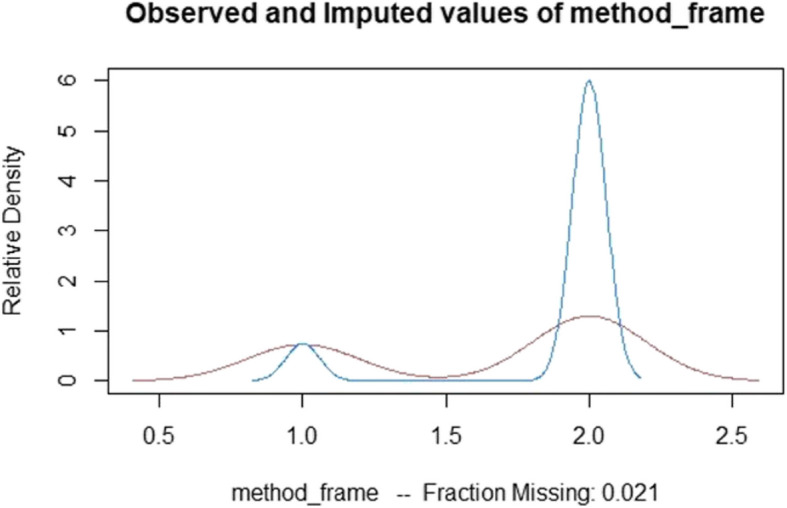
Distribution of VMMC method, comparing the raw values (blue) to imputed values (red)

## Data Availability

The datasets generated and/or analyzed during the current study are not publicly available. The data underlying the results presented in this study is Population Services International’s program data. Data can be requested from Population Services International’s research team, which can be reached at www.psi.org.

## Appendix 1

### Weighting summary

In this study, a random sample of patients from the facility records were selected for community verification, but not all patients were interviewed. Weighting the results therefore accounts for differences between the interviewed patients and all patients that were selected. Through six steps, inverse probability of treatment weighting (IPTW) was used. First, to determine which factors differentiated patients that were interviewed from patients that were not interviewed, t-tests comparing the two groups were conducted on all independent variables from Model 1. All variables were significantly different between the groups except for two: the number of trained clinical VMMC staff and the number of supervisory visits. Second, a logistic regression was run using the outcome of whether the patient was interviewed or not (1/0) and all independent variables that differentiated the two groups based on the first step. Third, predictions for the probability of being interviewed (prob) for each patient were then obtained based on the regression results. For the fourth step to increase the weight of patients that had low probabilities of selection, raw weights were calculated based on the inverse of the probability of being interviewed (1/prob). Fifth, the raw weights were stabilized by multiplying each weight by the proportion of patients that were interviewed among all selected patients. And finally, the weights were trimmed at 1th and 99^th^ percentiles to eliminate extreme outliers. Figure 3 shows the distribution of the weights at each step.

To check how well the weights corrected for bias, t-tests were again run but instead including the weights; about half of the independent variables were no longer significant between the patients that were interviewed and those that were not. To investigate further, the difference in means between the two groups were compared in the original data and the weighted data; comparing the two differences showed that the difference reduced for all variables. The results of these checks suggest the weights made the interviewed group more similar on average to the non-interviewed group. To further assess whether the interviewed group was adjusted appropriately relative to the entire unweighted sample, each variable was visualized such as with age in Fig. 4.

## Appendix 2

### Imputation summary

Amelia II in R software version 2022.12.0 (R Foundation for Statistical Computing, Vienna, Austria) was used for imputation. The patient-level dataset in this study had missing data for two facility-level variables (number of supervisory visits (*n* = 297) and the size of eligible population in catchment area (*n*= 42)) and one patient-level variable (VMMC method (*n* = 53)). First to impute the facility-level variables, 5 iterations were employed (*m* = 5) on a facility-level dataset using the facility-level variables that are in Model 1 (Table 1). These results were then merged into the patient-level dataset used in this study. To impute the VMMC method, 5 iterations were again employed (*m*= 5) on the patient-level dataset using the same variables as are in Model 1. Finally, the coefficients were combined in Stata using the mi command, which relies on Rubin's rules. Figure 5 presents the distribution of the raw data compared to the imputed values for the method.

## Appendix 3

**Table 5:** Description of patients in the complete case analysis

	Results with Imputed Values (*N*=2565)				Complete Case Analysis (*N*= 2,214)			
	Mean or Proportion	SD	Min	Max	Mean or Proportion	SD	Min	Max
Number of reported VMMCs	5658.2	3650.9	22.00	14829.0	5793.2	3776.4	22.00	14829.0
Number of staff	5.436	2.088	2	10	5.510	2.199	2	10
Number of gold mobilizers	4.239	0.991	1	6	4.290	1.038	1	6
Number of silver mobilizers	4.050	4.154	0	15	4.356	4.331	0	15
Number of vehicles	1.795	0.727	1	3	1.766	0.766	1	3
Population of catchment (Log)	8.782	1.017	6.380	11.96	8.589	0.835	6.380	11.96
Number of supervisory visits	2.532	2.048	0	10	2.492	2.024	0	10
Location distance (kilometers)								
[0]	0.412	0.492	0	1	0.343	0.475	0	1
[>0 to 50]	0.526	0.499	0	1	0.586	0.493	0	1
[>50 to 140]	0.0616	0.240	0	1	0.0714	0.257	0	1
Type of facility								
District	0.194	0.396	0	1	0.194	0.396	0	1
Mission	0.142	0.349	0	1	0.160	0.367	0	1
Rural clinic	0.562	0.496	0	1	0.579	0.494	0	1
Rural hospital	0.102	0.303	0	1	0.0664	0.249	0	1
Type of base funding								
Council	0.313	0.464	0	1	0.276	0.447	0	1
Government	0.465	0.499	0	1	0.472	0.499	0	1
Mission	0.207	0.405	0	1	0.235	0.424	0	1
Private	0.0140	0.118	0	1	0.0163	0.127	0	1
Method								
Prepex	0.111	0.314	0	1	0.110	0.313	0	1
Surgical	0.889	0.314	0	1	0.890	0.313	0	1
Verification round								
First	0.388	0.487	0	1	0.366	0.482	0	1
Second	0.612	0.487	0	1	0.634	0.482	0	1
Age of Patient								
Less than 15	0.374	0.484	0	1	0.384	0.487	0	1
15-29 years	0.583	0.493	0	1	0.582	0.493	0	1
30 or more	0.0421	0.201	0	1	0.0334	0.180	0	1

## Appendix 4

**Table 6:** Logit regression results for not being interviewed, stratified by catchment population and number of reported VMMCs (for Model 1)

Variables	Full Model	Stratified Analysis			
	Adj. Odds Ratio (*N*=2565)	Facilities with large catchment populations (*N*=1515)	Facilities with small catchment populations (*N*=1040)	Facilities that reported large numbers of VMMCs (*N*=1955)	Facilities that reported small numbers of VMMCs (*N*=610)
Number of reported VMMCs (Log)	1.38	1.60^*^	2.71	2.51^*^	1.70
Number of staff	1.13	0.85	1.27^*^	1.10	0.91
Number of gold mobilizers	0.83	1.00	0.88	0.85	1.19
Number of silver mobilizers	1.00	1.03	1.05	1.01	0.85
Number of vehicles	1.57	1.10	1.49	1.37	1.45
Population of catchment (Log)	0.99	0.65	0.50^**^	1.04	0.88
Location distance (kilometers)					
[0]	(base)	(base)	(base)	(base)	(base)
[>0 to 50]	0.62^*^	0.44^*^	0.95	0.59	0.65
[>50 to 140]	0.35^***^	0.13^***^	1.36	0.30^**^	0.28^*^
Number of supervisory visits	0.90	0.73^**^	1.06	0.96	0.93
Type of facility					
District	(base)	(base)	(base)	(base)	(base)
Mission	0.80	1.23	1.69	1.31	0.26
Rural clinic	1.07	0.99	0.25^*^	1.18	0.78
Rural hospital	1.46	5.93^***^	0.24	0.68	10.00^***^
Type of base funding					
Council	(base)	(base)	(base)	(base)	(base)
Government	1.10	1.64^*^	1.38	1.30	0.70
Mission	1.09	1.01	0.37	0.89	(omitted)
Private	0.46	3.46	0.35	0.21	0.42
Verification round					
First	(base)	(base)	(base)	(base)	(base)
Second	1.13	1.71^*^	2.93^*^	1.76*	1.11
Age of Patient					
Less than 15	(base)	(base)	(base)	(base)	(base)
15-29 years	2.92^***^	2.60^***^	3.31^***^	3.23^***^	2.05^**^
30 or more	2.10^**^	1.91^*^	2.29	2.48^**^	1.51
Method					
Prepex	(base)	(base)	(base)	(base)	(base)
Surgical	2.21^***^	1.95^**^	3.78^**^	2.57^***^	1.21
Constant	0.01^*^	0.84	0.00	0.00^***^	0.02
var(_cons[district])	1.59^*^	2.17	1.34	1.00	1.29
var(_cons[dis~t>par~e])	1.11	1.03	1.22	1.18	1.00
N	2565	1515	1040	1955	610

All odds ratios are adjusted by all the variables in the table

^*^*p* < 0.10

^**^*p* < 0.05

^***^*p* < 0.01

## Appendix 5

**Table 7:** Logit regression results for overreporting defined by when a patient did not plausibly confirm receipt, stratified by catchment population and number of reported VMMCs (Model 2)

Variable	Full Model	Stratified Analyses			
	Adj. Odds Ratio (*N* = 1412)	Facilities with large catchment populations (*N* = 763)	Facilities with small catchment populations (*N* = 634)	Facilities that reported large numbers of VMMCs (*N* = 1066)	Facilities that reported small numbers of VMMCs (*N* = 328)
Number of reported VMMCs (Log)	2.01^*^	1.34	2.66^*^	3.51^*^	1.92
Number of staff	1.02	1.21	1.12	0.97	0.95
Number of gold mobilizers	0.83	0.61	0.82^**^	0.81	1.19
Number of silver mobilizers	0.99	1.00	0.99	1.02	1.00
Number of vehicles	0.80	0.96	0.92	0.69	0.74
Population of catchment (Log)	0.92	1.36	1.25	0.82	1.06
Location distance (kilometers)					
[0]	(base)	(base)	(base)	(base)	(base)
[>0 to 50]	0.41^**^	0.34^**^	0.36*	0.16^**^	0.39^*^
[>50 to 140]	0.36^*^	0.37	0.56	0.09^**^	0.49
Number of supervisory visits	0.92	1.06	0.99	0.87	1.03
Type of facility					
District	(base)	(base)	(base)	(base)	(base)
Mission	1.30	1.63	0.67	1.66	0.12^**^
Rural clinic	1.68	3.17^*^	0.60	3.73	1.57
Rural hospital	3.34^*^	2.23	1.38	6.68^**^	4.57
Type of base funding					
Council	(base)	(base)	(base)	(base)	(base)
Government	0.90	0.47^*^	2.65	1.30	0.36
Mission	0.61	0.66	0.48	0.77	(omitted)
Private	0.27	(omitted)	1.35	0.68	(omitted)
Verification round					
First	(base)	(base)	(base)	(base)	(base)
Second	0.40^***^	0.25^***^	0.76	0.38^*^	0.43
Age of Patient					
Less than 15	(base)	(base)	(base)	(base)	(base)
15-29 years	2.21^***^	2.51^***^	1.62	1.98^**^	3.20^***^
30 or more	1.16	0.80	1.52	0.99	1.89
Method					
Prepex	(base)	(base)	(base)	(base)	(base)
Surgical	1.81	1.94	1.14	1.58	2.25
Interview Location					
On phone	(base)	(base)	(base)	(base)	(base)
In person	1.61^**^	1.47	2.07^*^	1.54^*^	1.89
Constant	0.00^*^	0.00^*^	0.00^**^	0.00	0.00
var(_cons[district])	1.48^*^	1.00	1.00	1.50	1.00
var(_cons[dis~t>par~e])	1.00	1.20	1.00	1.05	1.00

All odds ratios are adjusted by all the variables in the table

^*^*p* < 0.10

^**^*p* < 0.05

^***^*p* < 0.01

## Abbreviations

PBF: Performance Based Financing
VMMC: Voluntary Medical Male Circumcision
HMIS: Health Management Information System
MOHCC: Ministry of Health and Child Care
PSI: Population Services International
IPTW: Inverse Probability of Treatment Weighting
OR: Odds Ratio
CI: Confidence Interval
