# Supplementary material for: Factors associated with overreporting based on community verification results in a performance-based financing program in Zimbabwe
Source: BMC Health Serv Res. 2025 May 28;25:768. doi: 10.1186/s12913-025-12599-8 (PMC12121126; doi:10.1186/s12913-025-12599-8)
Supplement: Supplementary file 1 — Supplementary Material 1. [file 12913_2025_12599_MOESM1_ESM.pdf]

INTERVIEW GUIDE [DRAFT]

Data Quality Audit for the VMMC Programme

Understanding Challenges for VMMC Verifications of Performance Based Financing Programs

Reminders: Emphasize confidentiality from the informed consent & start recording.

**Interview details**	
Respondent ID	|______________________________|
Gender	☐ M ☐ F
Date of the interview	____ / ____ / 2020
Name(s) of the interviewer(s)	☐ Laura Chiota<br>☐ Trina Gorman

Module 1: Opening		
1	I see from our records that you worked for ____ of the four DQAs for this grant, totaling ____ surveys overall. Does that encompass all of your experience with DQAs?	☐ Yes → Q3<br>☐ No → Q2
2	What was your involvement in the other DQAs?<br>As we start the questions, remember that your answers can pertain only to the DQAs for the BMGF grant, which took place during _____.	[Free text response]
3	How would you rate yourself in terms of the amount of experience you have with being an enumerator – on a scale from 0 (no experience) to 10 (most experience)?	
4	~~To help orient us towards discussing the field work for the DQAs, can you start by telling us briefly about your role during the DQAs, who you worked with, and what a typical day look like during the home visit field work~~?<br>Change midway: To help orient us towards discussing the field work for the DQAs, can you start by giving us a 2 minute summary of your role during the DQAs	[Free text response]
**Module 2: Clients Found**		
We would like to spend time talking about the process of finding clients through home visits. To refresh your memory, clients were first called on the phone, and then sought in person if a phone interview wasn't possible.		
5	We mentioned before that you submitted ____ surveys. If you had to guess, among all clients that you were assigned, what percent were you able to interview? [Given an example if helpful to clarify]	|__|__|
6	As you think back, can you walk us through the broad steps that were followed to try to locate clients in **villages** & as you do so, list the members of the community/program/team that were involved in trying to locate clients.<br>[Probe: What role did the mobilizer play in finding clients? What did a typical interaction with the mobilizer look like from start to finish?]	[Free text response]

<table>
<tr><td>7</td><td>Thank you. Can you now do the same, but instead walk us through the broad steps of trying to locate clients in schools? As before, list the members that were involved.</td><td></td></tr>
<tr><td colspan="3">Thank you. That is really helpful. Now I’m interested to go through the reasons from the tracking sheets for when clients could not be found to learn how you and your colleagues defined them. We’d like you to answer this to the best of your knowledge based on what you remember about the protocols of your entire team. If you don’t know or aren’t sure, it’s no problem - just say so.</td></tr>
<tr><td>8</td><td>Can you describe typical examples of when a client was marked as not known in the community?</td><td>[Free text response]</td></tr>
<tr><td>9</td><td>Before marking a client as not known in the community, how many people would you talk with on average? It’s okay to give a range of numbers if one number doesn’t fit your memory, and if it depended on the scenario, describe the variation.</td><td>|Free text response]</td></tr>
<tr><td>10</td><td>In general, did you have sufficient time with the mobilizer, the village head, and other key people in the village? That is, were they available –not too busy with other priorities so were able provide the time you needed to find clients?</td><td>☐ Yes
☐ No</td></tr>
<tr><td>10.1</td><td>If not, why?</td><td></td></tr>
<tr><td>11</td><td>You mentioned that you talked with the village head (& other key people in the village [if that is true]). We are interested in your opinion of how likely it is that he/they would know everyone in their village. More specifically, complete the following sentence:
I would estimate that village heads & any others we typically asked would know whether a client is in their village ___ % of the time.
Thank you. So to be sure I understand, you think that on average there is a ___% chance that they were wrong, which would cause you to incorrectly marked the client as not known in the community.</td><td>|____|</td></tr>
<tr><td>11.1</td><td>Why did you choose |___________|?</td><td></td></tr>
<tr><td rowspan="2">12</td><td>I’m going to ask you about two other categories – “having insufficient contact information” & “having an invalid address”</td><td></td></tr>
<tr><td>Let’s start with the first. Can you describe typical examples of when a client was marked as “having insufficient contact information;” that is, the address was too vague.</td><td>[Free text response]</td></tr>
<tr><td>13</td><td>Can you describe typical examples of when a client was marked as “having an invalid address” or an address that did not make sense.</td><td>[Free text response]</td></tr>
<tr><td>14</td><td>[If worked in Gokwe North / Seke / Zvishavane]
I see that you worked in __________ district, which had higher rates of clients reported as __________. As you compare this district to other districts, what do you think could have influenced or caused these results? In what ways is this district different compared to other districts?
<table><tr><td>Gokwe</td><td>Unknown & Invalid</td></tr><tr><td>Seke</td><td>Unknown & Insufficient & Invalid</td></tr></table></td><td>[Free text response]</td></tr>
</table>

		Zvishavane	Unknown & Insufficient		

We know that there were some challenges that staff encountered in regards to the information on the clinical record – and we are interested in learning about your team's experiences with these challenges. We are going to read a scenario, and we would like you to rate how frequently this happened across the enumerators that you worked with during these DQA. So that it is easy for you to reference, do you have a pen and paper to write down the options briefly? They are: never, rarely, sometimes, often, always.

		Never→ Next Q	Rarely →B	Sometimes→B	Often→B	Always →B
15	~~The information provided on the clinical record described two or more people in an area, so it was not possible to know which person was really described on the clinical record.~~					
15.1	~~Can you tell me more about specific examples of this scenario – either from your experience or those of your colleagues? How did you narrow down?~~	[Free text response]				
16	Enumerators were suspicious that some information provided from the clinical record was made up. For example, the client's name, date of circumcision, age, method, or address.					
16.1	What made you (or other enumerators) suspicious?					
16.2	Can you tell me more about specific examples of this scenario – either from your experience or those of your colleagues?	[Free text response]				
17	Enumerators were suspicious that the information provided from the clinical record were about a fake/ghost client – that is, a client that does not exist.					
17.1	What made you (or other enumerators) suspicious?					
17.2	Can you tell me more about specific examples of this scenario – either from your experience or those of your colleagues?	[Free text response]				
18	So to summarize your answers, <briefly summarize>. Do you have ideas for how these scenarios could be avoided in future programs?<br>- Two or more people matched the information on a record<br>- Suspicious the record had some 'made up' data<br>- Suspicious the record was about a ghost client					

The questions above asked about the frequency of suspicion among enumerators. We are now interested in learning more about how sure or not sure you were that records were being fabricated in some way. So now focus instead on your own impressions and experiences instead of the entire team. We are going to go through three groups of people – clients, VMMC staff / clinicians, and the mobilizer – and we would like you to rate your certainty level on a scale from 0 (no suspicion) to 10 (indicating that you were certain data fabrication was happening). You can also answer 'undecided' if it's too hard to answer.

19	The first group are **clients**. On a scale from 0 to 10, what was your certainty level that some had provided false data on the clinical record?	

19.1	Why did you choose _____?	
20	The next group are the **VMMC staff/clinicians** who filled out the form. On a scale from 0 to 10, what was your certainty level that they had recorded false data on the clinical record?	
20.1	Why did you choose _____?	
21	Finally, the **mobilizer**. On a scale from 0 to 10, what was your certainty level that they had contributed false data about clients?	
21.1	Why did you choose _____?	

Module 3: **Acceptable Evidence**
Now I'd like to talk about the interviews with clients, both the home visits and the ones you did by phone. Just as before, I'm going to list some scenarios and I'd like you to estimate how frequently they occurred in these DQAs based on your experience. Unlike the previous set of scenarios, these are based on your own experience alone – not your impression of the entire team. The possible answers are the same: never, rarely, sometimes, often, always.

		Never → Next Q	Rarely →B	Sometimes →B	Often→B	Always →B
First, we are interested in whether there were negative unintended consequences of doing this work. By "negative unintended consequences" I mean negative challenges/problems that occurred because of the DQAs that were not foreseen.						
22	While both making phone calls and looking for clients during home visits, I witnessed times when these efforts caused negative feelings or strong resistance from spouses.					
22.1	Can you describe an example of this scenario?					
23	While both making phone calls and looking for clients during home visits, I witnessed times when these efforts caused negative feelings or strong resistance from the clients.					
23.1	Can you describe an example of this scenario?	[Free text response]				
24	While both making phone calls and looking for clients during home visits, I had confidentiality concerns whereby a client's circumcision status could have been suspected/known by people other than himself (and the guardian that signed the clinical record, if a minor). As an example, community members could have suspected that enumerators were looking for VMMC clients if they saw enumerators with mobilizers – given mobilizers promoted VMMC in the past. Did you have these concerns or other confidentially concerns for other reasons – Never, Rarely, Sometimes, Often, or Always?	[Free text response]				
24.1	Can you describe an example of this scenario?	[Free text response]				

25	Do you think these issues impacted the quality of the survey data – for example, it reduced the portion of clients that were found and/or the quality of the survey data? Why or why not?	[Free text response]
	Next, again we know that this work is challenging and clear information is not always readily available. Our next statement is…	
26	I completed/submitted an interview with a respondent even when I was not certain that the respondent was the correct person.	
26.1	Can you describe an example of this scenario?<br>[Probe: What made you think the client might not be the correct person? What questions did you ask, and who did you talk to about the issue? Did you finish the interview?]	[Free text response]
27	While interviewing a client, I had the feeling that the person was not being truthful.	
27.1	Can you give an example of this scenario?	[Free text response]
28	I was interviewing a client and he (or the parent) reported not being circumcised.	
28.1	Can you describe an example of this scenario?	[Free text response]
28.2	What is your best guess of what happened? Why?	[Free text response]
29	Some clients did not meet acceptable evidence because the reported method (surgical, prepex) did not match the data captured from the clinical record. We know there were times when some clients did not initially understand or know the words 'surgical' or 'prepex' so might have needed the enumerator to explain. But after any explanation – at the time when the client answered the question – do you think it's possible that some clients still did not know what each of the methods were? Why or why not?	

<table>
<tr><td colspan="3">Now we are interested in brainstorming ideas regarding what could influence the extent to which a client is truthful with an enumerator about this sensitive topic. Your responses don't need to be from actual experiences, but you can speculate based on what you have heard or know about the communities that you work in and their cultural norms and attitudes.</td></tr>
<tr><td>30</td><td>Can you think of any reasons why clients who were in fact circumcised would report that they were not?</td><td>[Free text response]</td></tr>
<tr><td>31</td><td>Can you think of any reasons why clients who were not in fact circumcised would report that they were?</td><td>[Free text response]</td></tr>
<tr><td>32</td><td>In future VMMC programs, what do you think might be done to increase the portion of clients that are found and/or improve the field work?</td><td>[Free text response]</td></tr>
<tr><td>33</td><td>Is there anything that didn't come up today, that you think we should know about the client follow-up work?</td><td></td></tr>
<tr><td colspan="3">That completes the questions we had for you today. Do you have any last questions for us before we close the interview? Once again, thank for your time.</td></tr>
</table>

Module 4: Patients that Reported not being Circumcised		
**Client Sampling**		
1	Can you walk us through the brief steps that were followed to sample clients who would be followed up during the home visit exercise? As you do, mention if any different protocols were taken from specific DQAs if you were involved in more than one.	[Free text response]
2	[If they don't mention] There was a period where incomplete CIRs were sampled. Do you recall this?	☐ Yes<br>☐ No
3	If yes, which DQA was it for, and what was your process?	[Free text response]
4	Did you remember anyone you worked with ever selecting an incomplete CIR and not setting it aside to a separate pile / list of names?	[Free text response]
**Incomplete VMMCs**		
One of the scenarios we are trying to understand more is when clients reported not being circumcised during their interview. In our interviews with enumerators, one typical example we heard is that some clients reported that they signed up but didn't go through with the procedure, often because they were scared of the pain. Other reasons were that the client's family didn't agree, or the VMMC team didn't come back to pick the client. We are trying to understand what happened in terms of the completion of the CIRs for such clients at the site?<br><br>To orient us to the general program, the overall process was that mobilizers founds clients and then came back to collect consent forms & set up logistics like the date/transportation. Then at least the driver came to pick up clients—so would have known who to pick up & where. The VMMC location was sometimes an outreach site (roving teams) & other times was at a larger health facility.		
5	Do you know how much information / data was collected before clients came on the day of the procedure?<br><br>Probing questions: Did mobilizers or the drivers have:<br><br>a) Their address, phone number, and age – or instead<br><br>b) Only more minimal information such as 1) the consent form, and 2) the location/day of pickup?	[Free text response]
Thank you. So back to this scenario where there is a completed CIR for a client who didn't complete a procedure. Of all the fields on the CIR, the fields that stand out as most puzzling are fields like "HIV test offered", "Circ Name", "Discharged signature" and "Date of Device Placement". This isn't information that would have been available to the mobilizer when he picked up the consent form.		
6	These fields could have been filled in a way that was <u>intentional fraud</u>, or <u>unintentional</u> due to bookkeeping problems. We are going to talk about each, but let's start with unintentional bookkeeping problems. One example scenario that we have heard is this: that perhaps this happened during outreach where a lot of clients needed to be circumcised. If the roving teams of clinicians were working off of a register of client names, originally provided by the mobilisers, and were too busy to fill out the CIR in the field, then maybe they filled the CIR back at their clinics & simply assumed all clients were circumcised.<br><br>Do you have any other example scenarios that could have explained this – where the clinicians were <u>not</u> doing it intentionally? For example, are types	[Free text response]

	of sites / staff / locations / conditions that create a convincing explanation?	
7	Next let's again brainstorm scenarios but instead for intentional fraud. That is, when clinicians purposely filled in the data even though they knew the client was not circumcised. This could have happened if they tracked client attendance the day of the VMMCs but filled the forms anyway, knowing they would be paid more & assuming they wouldn't get caught. Another example is in the later DQAs when the clinicians knew what fields were important for the DQA, they may have filled in the empty important fields as a guess to make sure they got paid for the CIR.<br><br>***Probes:***<br><br>Are there other example scenarios that you think could have explained this? For example, are types of sites / staff / locations / conditions that create a convincing explanation?	[Free text response]
8	Next, we are curious what you think happened most often. In this scenario where there were complete forms for incomplete VMMCs—if you had to guess—do you think it was most often intentional, or unintentional bookkeeping issues? Its okay to say you aren't sure.	☐ intentional<br>☐ unintentional<br>☐ does not know
9	Do you have any other thoughts to share that you haven't mentioned already?	[Free text response]
